# Multimorbidity Patterns and Depression: Bridging Epidemiological Associations with Predictive Analytics for Risk Stratification

**DOI:** 10.3390/healthcare13121458

**Published:** 2025-06-18

**Authors:** Xiao Wang, Nan Zheng, Mei Yin

**Affiliations:** 1 School of Medical Humanity, Harbin Medical University, Harbin 150081, China; wangxiao@hrbmu.edu.cn; 2 Continuing Education Department, Harbin Medical University, Harbin 150081, China; 102453@hrbmu.edu.cn

**Keywords:** multimorbidity patterns, depression, latent class analysis (LCA), XGBoost

## Abstract

**Background:** Late-life depression is a critical public health concern, particularly among older adults with chronic multimorbidity. Existing studies often focus on single-disease associations, neglecting the complex interplay of coexisting conditions. Understanding how multimorbidity patterns contribute to depression risk and identifying high-risk subgroups through integrated statistical and machine learning approaches remain underexplored, limiting targeted prevention strategies. **Methods:** Using data from the China Health and Retirement Longitudinal Study (CHARLS), latent class analysis (LCA) was employed to cluster multimorbidity patterns. Associations between these patterns and depression were analyzed using multivariable logistic regression, while predictive performance and interaction effects were evaluated via an XGBoost machine learning model. **Results:** Four distinct multimorbidity patterns were identified: cardio-metabolic, digestive–joint, respiratory, and cardiovascular–digestive pattern. All clusters showed significant independent associations with depression, with the cardiovascular–digestive pattern exhibiting the strongest association (OR = 4.56). However, the digestive–joint pattern demonstrated the highest predictive effects for depression. Sociodemographic factors—low income, limited education, female gender, and rural residence—emerged as robust predictors, amplifying depression risk in older adults with multimorbidity. **Conclusions:** This study bridges epidemiological insights with predictive analytics to inform depression risk stratification. We recommend routine depression screening for all individuals with cardiovascular–digestive diseases and prioritize screening for women with digestive–joint diseases. Additionally, low-income and rural-dwelling older adults with chronic conditions warrant heightened clinical vigilance. These findings provide a framework for integrating multimorbidity profiling into depression prevention protocols, addressing both biological and socioeconomic determinants.

## 1. Introduction

Depression constitutes a major contributor to the global disease burden, characterized by core biopsychosocial manifestations encompassing diminished self-worth, sleep–wake cycle disturbances (e.g., early morning awakening or hypersomnia), appetite dysregulation (including hyperphagia or anorexia), cognitive impairment in concentration, and recurrent death ideation [1]. As China’s aging population grows rapidly, depressive disorders among older adults have turned into a critical public health concerns. Data from a national survey shows that 34.1% of Chinese older adults exhibit depressive symptoms, with the prevalence among females reaching 41.5% [2]. The harms associated with depression in old age have serious implications for individual health, family functioning, and socioeconomics. Empirical evidence suggests that the risk of all-cause mortality in middle-aged and older adults with depressive symptoms is 20% higher than in those without [3]. Furthermore, geriatric depression frequently coexists with social withdrawal and diminished activities of daily living, substantially intensifying caregiver burden and public healthcare expenditures [4,5,6]. Accumulating evidence highlights the critical need for accurate depression risk stratification in older adults. Early identification helps to detect potential depression problems in a timely manner and effectively improve the physical and mental health of older persons through targeted interventions, such as psychotherapy, medication, and social support.

Notably, the pathogenesis of depression in middle-aged and older adults is intricately intertwined with multimorbidity (defined as the coexistence of two or more chronic diseases), forming a complex pathophysiological network. A study based on the Fourth National Sample Survey of the Elderly Population in Urban and Rural China shows that the prevalence rate of chronic diseases among senior citizens in China reaches 50.5%, and the multimorbidity rate increases with age, ranging from 39.4% to 53.6% as a whole [7]. The intricate pathological interactions inherent to multimorbidity not only accelerate physical functional decline, but also establish bidirectional vicious cycles with depression through neuroinflammatory pathways (e.g., elevated IL-6 and CRP levels) and psychosocial stressors (e.g., increased medical burdens and deficient social support) [8]. Research in China has demonstrated that both individual chronic diseases and multiple chronic conditions significantly increase depression scores, establishing multimorbidity as a critical determinant of depression among older adults [9]. Similarly, an investigation in South Korea has observed elevated depression prevalence corresponding to the presence and number of comorbidities, with particularly pronounced symptoms among those experiencing four or more chronic conditions [10]. Further evidence indicates that specific disease dyads—particularly combinations of cardiovascular, gastrointestinal, and chronic respiratory diseases—exert disproportionate effects on depression trajectories [11]. Despite these advances, extant studies predominantly quantify multimorbidity through simplistic disease counts or pairwise combinations, overlooking pathophysiological clustering patterns. Crucially, research examining associations between clinically coherent multimorbidity constellations and depression remains scarce, despite its pivotal implications for early intervention. Such investigations could empower clinicians to implement targeted preventive strategies against depression progression while elucidating underlying mechanistic pathways.

To address these gaps, this study introduces a multi-method framework that integrates latent class analysis (LCA), epidemiological modeling, and explainable machine learning to decode depression risk mechanisms using the 5th wave of CHARLS data (N = 17,424). A three-phase analytical scheme was implemented: first, LCA was used to identify mutually exclusive subgroups of multimorbidity based on chronic disease profiles. Second, multivariate logistic regression adjusted for sociodemographic and behavioral confounders was performed to validate independent associations between patterns and depression. Third, XGBoost modeling with SHAP value interpretation was employed to quantify predictive hierarchies and interaction effects of multimorbidity combinations on depression risk. This study was designed to elucidate the relationship between multimorbidity patterns and depression, allowing for the proposal of stratified and targeted intervention and screening protocols.

## 2. Materials and Methods

### 2.1. Study Population

Data for this study were from the China Health and Retirement Longitudinal Study (CHARLS), a nationally representative longitudinal survey targeting individuals aged 45 years and older in China. By strictly implementing stratified probability proportional to size (PPS) random sampling and maintaining rigorous quality control, the survey sample effectively represents the targeted population in China. CHARLS collects multidisciplinary data relating to basic demographics, socioeconomic status (SES), and health information of the participants. Its baseline data collection began in 2011 with 17,708 participants, followed by follow-up waves in 2013, 2015, 2018, and 2020, achieving retention rates exceeding 86% across all waves. Data collection of the survey was ethically approved by the Peking University Biomedical Ethics Review Board (IRB00001052-11015), and written informed consent was obtained from all participants [12]. The data used in this study were applied for and obtained by Wang through the official website of CHARLS.

The current study drew on data collected in 2020, including 19,395 participants, to present the latest insights into the predictive role of multimorbidity in depression. Participants with missing data for basic demographic information (*n* = 111), any of the 15 chronic diseases (*n* = 32), and depressive symptoms (*n* = 1828) were excluded; a final sample of 17,424 was included in the investigation. There are no outliers in the dataset.

### 2.2. Assessment of Depression

Depressive symptoms were evaluated using the 10-item Center for Epidemiological Studies Depression Scale (CES-D), a modified version of the original 20-item full-length CES-D [13]. The tool has demonstrated good reliability and validity for assessing the mood and behavioral patterns related to depressive symptoms among the Chinese elderly. The participants were surveyed through the questionnaire about the frequency of experiencing the following 10 feelings within the past seven days: (1) being bothered by trivial matters; (2) having difficulty concentrating; (3) feeling down in the dumps; (4) finding it laborious to perform any tasks; (5) being full of hope for the future; (6) feeling fearful; (7) having trouble sleeping well; (8) being in a cheerful mood; (9) feeling lonely; and (10) feeling that they could not go on with life. The options are “Rarely or not at all (less than 1 day)”, “Not very often (1–2 days)”, “Sometimes or about half of the time (3–4 days)”, and “Most of the time (5–7 days)”, with scores ranging from 0 to 3 points. For the 2 questions reflecting positive emotions (“being full of hope for the future” and “being in a cheerful mood”), the scores were assigned in the opposite way. The final score was obtained by adding up the scores of the 10 questions. The total scores of each participant ranged from 0 to 30 points, and those with scores of 10 points or above were assessed as having depression.

### 2.3. Assessment of Multimorbidity

In CHARLS, the presence of chronic diseases among participants is ascertained via the following question: “Have you been diagnosed with any of the following diseases by a doctor?” The response options consist of 15 chronic disease types, including hypertension, dyslipidemia, diabetes, cancer, chronic lung disease, chronic liver disease, chronic heart disease, stroke, kidney disease, stomach disease, emotional problems, memory-related disease, arthritis, asthma, and Parkinson’s. In this study, individuals suffering from two or more chronic diseases were identified as participants with multimorbidity.

### 2.4. Covariates

The covariates included demographic and socioeconomic data, such as gender (male, female), age (45–59 years, 60–69 years, ≥70 years), education level (uncompleted primary education, primary school, secondary school, senior high school or above), marital status (married, others including divorced, widowed and never married), residence (rural, urban), and annual household income level (lowest; second-lowest; medium; second-highest; highest). Behavior factors were also considered as covariates, including smoking status (current smoker; non-smoker), alcohol consumption (current drinker; non-drinker) and physical activity (physically active; physically inactive). In the following multivariable logistic regression analysis, these covariates were adjusted as potential confounders to identify independent statistical associations between multimorbidity patterns and depression, whereas in the XGBoost machine learning framework, they were incorporated as predictive features to maximize model discrimination accuracy.

### 2.5. Statistical Analysis

Baseline characteristics of participants were summarized using descriptive statistics, with categorical variables presented as frequency counts and percentages. Between-group comparisons were conducted using Pearson’s chi-square tests.

Latent class analysis (LCA) is a person-centered statistical approach that identifies distinct subgroups within a population based on their observed response patterns to categorical indicators. LCA aims to uncover latent structures by grouping individuals with similar response patterns, thereby maximizing homogeneity within each subgroup while maintaining heterogeneity between subgroups. The core objective of this method is to derive the most parsimonious model that maximizes within-class homogeneity while ensuring between-class heterogeneity, thereby revealing latent structures underlying complex multivariate data. Using the Expectation–Maximization (EM) algorithm, the model iteratively estimated two key parameters: class membership probabilities, representing the proportion of the population assigned to each latent class, and item–response probabilities, quantifying the likelihood of disease manifestation within each class. This study employed latent class analysis (LCA) to identify multimorbidity patterns among 17,424 participants, with 15 chronic diseases coded as binary variables (presence = 1, absence = 0). Models specifying 1 to 6 latent classes were systematically constructed and evaluated using established fit indices, including the Akaike Information Criterion (AIC) given by Equation (1), Bayesian Information Criterion (BIC) given by Equation (2), and adjusted BIC (aBIC). AIC measures model fit while penalizing complexity, with lower values indicating better parsimony; BIC similarly penalizes complexity but with a stronger emphasis on sample size, favoring models that balance fit and theoretical simplicity; aBIC adjusts BIC for smaller samples, providing a more conservative criterion for class selection.

(1) AIC=2k−2lnL

where *k* represents the number of parameters and *L* represents the logarithmic likelihood.

(2) BIC=klnn−2lnL

where *n* represents the sample size.

Optimal class selection requires lower values of AIC, BIC, and aBIC, alongside higher entropy values *E* to ensure clear class separation, which is represented as Equation (3). Additionally, the bootstrap likelihood ratio test (BLRT) was used to evaluate whether adding classes significantly improved model fit (*p* < 0.001), ensuring that the final model was statistically justified. When selecting the final model, the sample size of each group should meet the minimum proportional requirement (≥4%) to avoid parameter estimation bias.

(3) $$E=-\sum p_{k}\log_{2}p_{k}$$

where $p_{k}$ represents the probability of belonging to the *k*th class.

Before clarifying the associations between multimorbidity patterns and depression, univariate and multivariate logistic regression analyses were first conducted to verify the association between the presence of multimorbidity (i.e., having at least two chronic diseases vs. having none or only one chronic disease) and depression, aiming to learn the relationship between multimorbidity and depression in the first place (statistical significance was determined with *p* < 0.05). Next, taking the multimorbidity patterns classified by latent class analysis (LCA) as independent variables (with the relatively healthy class as the reference group), univariate logistic regression was used to preliminarily analyze the associations between different multimorbidity patterns and depression. Three hierarchical multivariable logistic regression models were constructed to refine these associations through sequential adjustment for confounders. Model 1 adjusted for no covariates to estimate unassisted associations. Model 2 controlled for core structural confounders, including gender, age, education level, marital status, residence, and annual household income. Model 3 further incorporated behavioral covariates, including smoking status, alcohol consumption, and physical activity. This tiered approach was designed to distinguish the incremental effects of sociodemographic factors from those of behavioral factors while mitigating overfitting through the inclusion of parsimonious covariates. The variance inflation factor (VIF) was used to evaluate the multicollinearity among independent variables in the model. Variables with a VIF > 5 and without clear clinical significance were removed to address the multicollinearity issue. Finally, the independent associations between multimorbidity patterns and depression was determined through the multivariate model. The results were presented as odds ratios (ORs) and their 95% confidence intervals (CIs), and a *p*-value < 0.05 was considered statistically significant.

To further delineate the nonlinear associations between multimorbidity patterns and depression, analyze their interaction effects with concomitant risk factors, and investigate the moderating effects of multimorbidity prevalence on predictive contributions, the XGBoost algorithm was implemented to develop a depression risk prediction framework targeting middle-aged and older populations, prioritizing assessment of multimorbidity pattern contributions. The XGBoost algorithm minimizes a composite objective function that balances a binary cross-entropy loss term (measuring prediction error) with regularization penalties (L1/L2 norms on tree weights and a penalty on tree complexity). The model was constructed iteratively, with each new tree fitted to the pseudo-residuals (negative gradients of the loss function) from the previous iteration, leveraging both first- and second-order gradients to capture the curvature of the loss landscape. Categorical predictors underwent factorization, whereas ordinal variables such as educational attainment and income quintiles retained their natural hierarchical ordering. A stratified sampling approach ensured balanced outcome distribution, allocating 80% of observations to model training and 20% to validation. Model specification included the following: logistic loss function optimization with adaptive learning rate (η = 0.1), constrained tree architecture (max depth = 6), and stochastic regularization via feature/instance subsampling (both at 80%). Iterative boosting (1000 rounds) incorporated early termination protocols (50-round patience threshold) to curb overfitting. Predictive interpretation leveraged both gain-based feature importance and SHAP value decomposition, specifically isolating multimorbidity pattern effects. SHAP (Shapley Additive Explanations) provides a systematic way of interpreting model predictions by quantifying how much each feature contributes to an individual’s predicted depression risk. Unlike traditional methods that assume linear relationships, SHAP accounts for nonlinear interactions and social determinant modifiers. Positive SHAP values for a multimorbidity class indicate increasing depression probability, and negative values suggest protective associations. This allows for the identification of the patterns that drive depression most strongly and how contextual factors alter their effects.

Nonlinear associations and comorbidity–social determinant interactions were visualized through SHAP partial dependence plots. A comprehensive evaluation encompassed discriminative capacity (AUC-ROC, sensitivity/specificity balance) and probabilistic calibration (Brier score metrics).

Descriptive statistics and logistic regression analyses were conducted using SPSS (version 29.0.2.0). Latent class analysis (LCA) was performed with Mplus (version 8.0). The XGBoost model development, validation, and SHAP interpretation were implemented in R (version 4.3.3) using specialized packages, including xgboost for model construction and SHAPforxgboost for interpretability analysis. A two-tailed *p*-value < 0.05 was considered statistically significant. To control for type I error inflation from multiple comparisons, we applied the Benjamini–Hochberg false discovery rate (FDR) correction to all primary association analyses, with statistical significance redefined as *q* < 0.05. Interaction analyses utilized Bonferroni correction, where significance thresholds were set at α = 0.05, divided by the number of tested interactions.

## 3. Results

### 3.1. Baseline Characteristics

This study included a total of 17,424 participants aged 45 years and above, among whom 8141 were male (46.72%) and 9283 were female (53.28%). In terms of age, 7207 participants (41.36%) were aged 45–59 years, 5836 (33.50%) were aged 60–69 years, and 4381 (25.14%) were aged 70 years and above. The majority of participants were characterized as married (85.41%), having a primary education or below (64.62%), and residing in rural areas (60.54%).

The sample demonstrated substantial chronic disease burden with an overall prevalence rate of 80.7%, including 58% with multimorbidity (≥2 chronic conditions). The three most prevalent chronic diseases were hypertension (39.4%), arthritis (39.0%), and gastric disorders (32.0%).

During the 2020 survey period, the depression prevalence among Chinese older adults was 37.6%. Statistically significant differences in depression rates were observed across subgroups stratified by age, sex, marital status, educational attainment, residential location, physical activity, smoking status, alcohol consumption, income level, chronic disease comorbidity status, and comorbidity patterns (all *p* < 0.05), as detailed in Table 1.

### 3.2. Latent Classes of Multimorbidity Patterns

In this study, latent class analysis (LCA) was applied to examine comorbidity clustering patterns across 15 chronic diseases. Models with one to six classes were sequentially evaluated. As shown in Appendix A, the Akaike Information Criterion (AIC), Bayesian Information Criterion (BIC), and adjusted BIC (ABIC) values progressively decreased from the 1-class to 6-class models. Both the Lo–Mendell–Rubin-adjusted likelihood ratio test (LMR-LRT) and bootstrap likelihood ratio test (BLRT) indicated statistically significant improvements (all *p* < 0.001). Although the 6-class model demonstrated the lowest AIC (174,950) and BIC (175,688) values, reflecting optimal statistical fit, its clinical utility was constrained by over-segmentation. Specifically, one subgroup accounted for only 1.2% of the total sample (*n* = 220), which lacks epidemiological significance and poses challenges for targeted interventions. Moreover, stability analysis through 100 random 80% subsamples revealed high class assignment consistency for the 5-class structure.

When considering the model complexity, practical application value, and overfitting risk, the 5-class model was finalized as the optimal solution. Although its AIC (175,730) and BIC (176,343) values were slightly higher than those of the 6-class model, the 5-class model effectively mitigated the risk of overfitting. Additionally, its classification accuracy remained within an acceptable and reasonable range. Results from likelihood ratio tests (LMR-LRT/BLRT: *p* < 0.001) indicated that the 5-class model exhibited significant advantages over simpler models.

**Figure 1:**
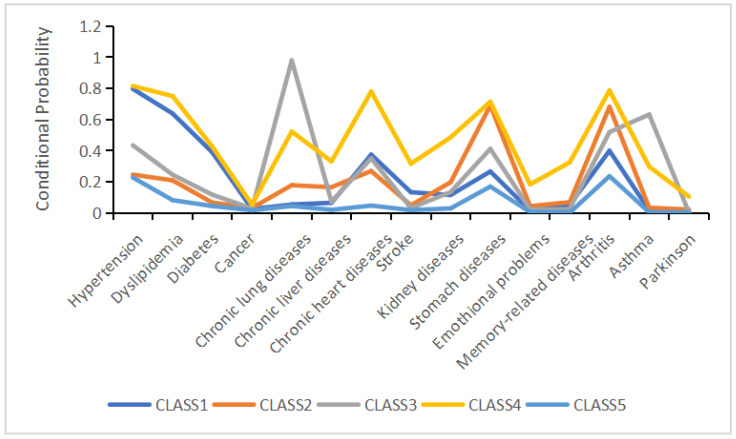
Five-class model of multimorbidity patterns.

Based on the distinct conditional probability profiles of the five classes across 15 chronic diseases as shown in Figure 1, we named class 1 the “cardio-metabolic class,” class 2 the “digestive–joint class,” class 3 the “respiratory class,” class 4 the “cardiovascular–digestive class,” and class 5 the “relatively healthy class.” The relatively healthy class was the largest subgroup, comprising 53% of the total sample (*n* = 9234). Participants assigned to the cardio-metabolic, digestive–joint, cardiovascular–digestive, and respiratory classes accounted for 21.7% (*n* = 3779), 16.3% (*n* = 2835), 4.8% (*n* = 838), and 4.2% (*n* = 738) of the total sample, respectively.

In the cardio-metabolic class, the prevalence of hypertension and dyslipidemia was 86.8% and 70.4%, respectively. The digestive–joint class exhibited high prevalence of gastric diseases (86.5%) and arthritis (78.2%). The respiratory class had a 100% prevalence of lung diseases, with asthma affecting 79.4% of this subgroup. In the cardiovascular–digestive class, the prevalence of hypertension, heart disease, and gastric diseases were 83.9%, 82.5%, and 76.1%, respectively. Participants without chronic comorbidities and those at low risk of comorbidity were classified into the relatively healthy class.

### 3.3. Associations Between Multimorbidity Patterns and Depression

Multivariate logistic regression analysis revealed a significant association between multimorbidity and depression as shown in Appendix B. After adjusting for sociodemographic variables (age, sex, education, etc.) and behavioral factors such as smoking and alcohol consumption, multimorbidity independently increased the risk of depression (OR = 2.156, 95% CI: 2.014–2.309). Further analysis using multivariate logistic regression identified significant associations between distinct multimorbidity patterns and depression as shown in Table 2. Taking the relatively healthy class (C5) as the reference group, unadjusted logistic regression (Model 1) demonstrated statistically significant elevations in depression risk across all comorbidity classes. Specifically, the cardio-metabolic class (C1) exhibited an odds ratio (OR) of 1.61 (95% CI: 1.49–1.74), followed by the digestive–joint class (C2; OR = 2.43, 95% CI: 2.23–2.64), the respiratory class (C3; OR = 2.24, 95% CI: 1.93–2.61), and the cardiovascular–digestive class (C4), which displayed the highest risk (OR = 4.74, 95% CI: 4.08–5.51).

Upon adjustment for sociodemographic covariates (gender, age, education level, residential area and marital status), partial attenuation of effect estimates was observed: the OR for C1 marginally increased to 1.65 (95% CI: 1.52–1.80), whereas ORs for C2, C3, and C4 decreased to 2.25 (95% CI: 2.06–2.46), 2.20 (95% CI: 1.88–2.57), and 4.58 (95% CI: 3.92–5.36), respectively, with all associations retaining statistical significance (*p* < 0.001). Subsequent inclusion of behavioral confounders (smoking, alcohol consumption, physical activity and income level) further stabilized the OR estimates: C1, 1.65 (95% CI: 1.51–1.80); C2, 2.24 (95% CI: 2.05–2.45); C3, 2.19 (95% CI: 1.87–2.56); C4, 4.56 (95% CI: 3.90–5.34). Confidence intervals remained exclusive of the null value (1.00), and all *p*-values persisted below 0.001.

Notably, the cardiovascular–digestive multimorbidity class (C4) retained a 4.56-fold increased depression risk compared to the healthy class in Model 3, significantly higher than other comorbidity patterns. The minimal attenuation in effect sizes across models indicated that sociodemographic and behavioral factors only partially explained the associations, suggesting that the impact of multimorbidity on depression primarily stems from its independent effects.

### 3.4. Xgboost Model Performance

Following hyperparameter optimization and class weighting, the XGBoost model developed in this study demonstrated robust performance and clinical utility in depression risk prediction. The model achieved an overall accuracy of 64.72% (95% CI: 0.6311–0.6631) on the test set, significantly exceeding the no-information rate (*p* = 0.002, α = 0.05); this indicates statistically meaningful predictive capacity. Balanced sensitivity (64.34%) and specificity (65.37%) yielded a balanced accuracy of 64.85%. The discriminative ability was further validated by the receiver operating characteristic (ROC) curve, with an area under the curve (AUC) of 0.694. The AUC of 0.694 remains clinically informative in the context of depression screening. Given the multifactorial nature of depression and the scarcity of validated biomarkers, an AUC of 0.694 demonstrates moderate discriminative ability that could aid clinicians in identifying at-risk individuals when combined with clinical judgment. In terms of classification performance, the F1-score (0.694) reflected the model’s balanced capability for high-risk population screening, suggesting an optimal trade-off between precision (PPV = 75.49%) and recall (sensitivity = 64.34). Such performance supports its utility as a first-stage screening tool that could be integrated into routine primary care visits, particularly for identifying high-risk subgroups with complex multimorbidity patterns who may benefit from early psychosocial interventions.

### 3.5. Predictive Value of Multimorbidity Patterns on Depression

**Figure 2:**
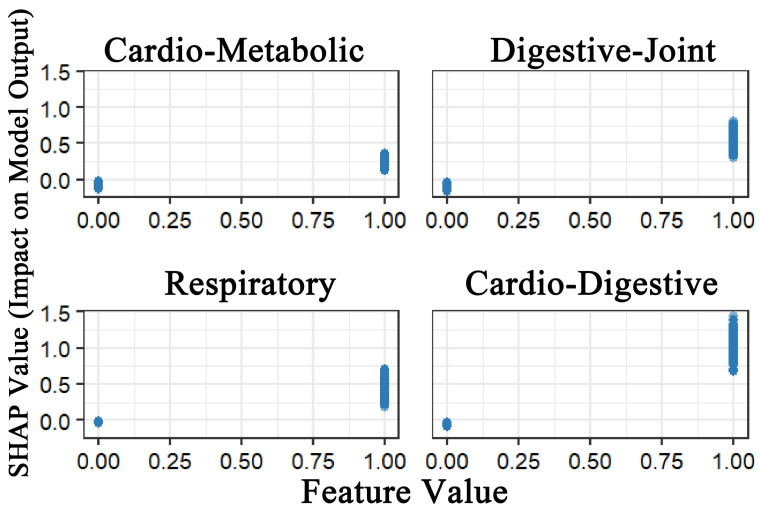
SHAP value impact of four multimorbidity patterns on model output.

To address the black-box nature of machine learning models, SHAP was applied to interpret the XGBoost model. Figure 2 demonstrated that all four multimorbidity patterns exhibited positive predictive effects on depression. Among these, the Class 4 multimorbidity pattern (cardiovascular–digestive cluster) exerted the strongest predictive effect, followed by Class 2 (digestive–joint cluster). While Class 1 (cardio-metabolic cluster) and Class 3 (respiratory cluster) also showed predictive utility, although their effects were relatively weaker. This finding demonstrates strong consistency with the results from the logistic regression analysis.

**Figure 3:**
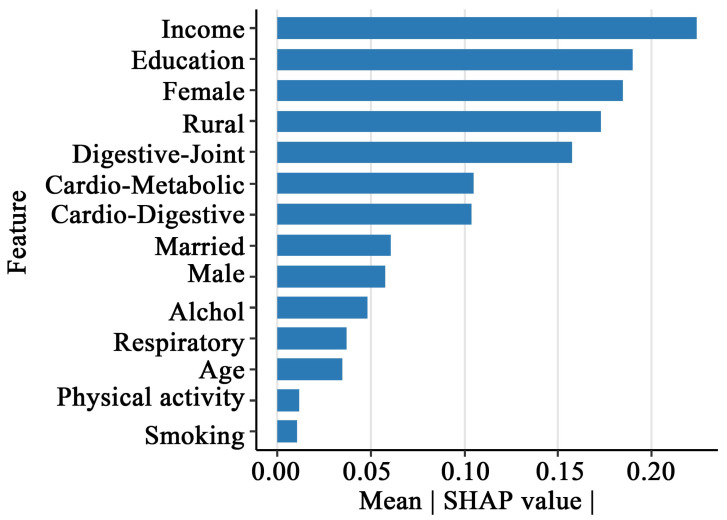
Feature importance plot of the XGBoost model.

Figure 3 illustrates the global feature importance for depression prediction. Results revealed that all four multimorbidity patterns served as significant predictors of depression. Notably, Class 2 demonstrated the highest mean SHAP value, indicating its broad and statistically substantial influence across the model. Class 1 and Class 4 exhibited comparable mean SHAP values, and Class 3 displayed the least influential contribution. The model also identified key sociodemographic predictors, with income level, educational attainment, gender, and residence emerging as the most critical determinants of depression risk.

**Figure 4:**
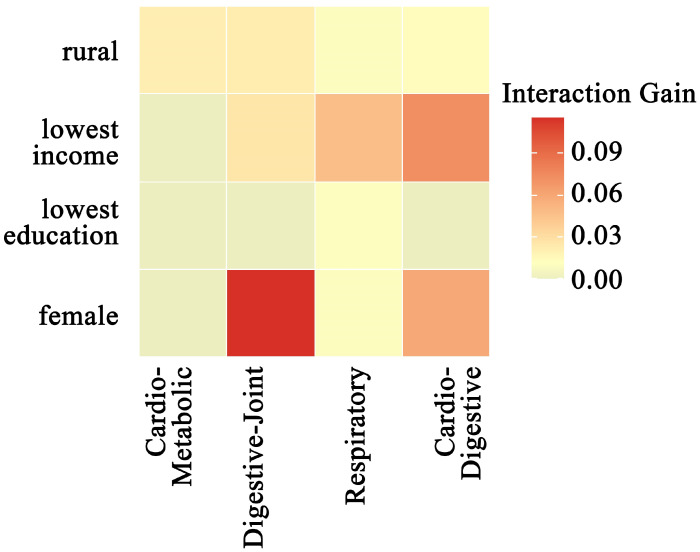
Heatmap of interaction gain between multimorbidity patterns and sociodemographic factors.

Interaction analysis (Gain > 0.01) revealed significant synergistic effects between the four multimorbidity patterns and multiple sociodemographic variables (Figure 4). Among these, the interaction between the Class 2 pattern (digestive–joint cluster) and female sex exhibited the most substantial contribution to model predictions (Gain = 0.115). The Class 4 pattern (cardiovascular–digestive cluster) demonstrated particularly complex interaction effects, with significant synergies observed for lowest-income levels (Gain = 0.072) and female sex (Gain = 0.059). Notably, lowest income and rural residency both showed significant interactions with most multimorbidity patterns.

Following Benjamini–Hochberg FDR correction, all previously significant associations between multimorbidity patterns and depression retained statistical significance (*q* < 0.001). For interaction analyses, after Bonferroni correction (α = 0.003125 per test, family-wise α= 0.05), all interactions discussed above remained significant.

### 3.6. Sensitivity Analysis

To evaluate the robustness of the association between multimorbidity patterns and depression, we conducted propensity score matching (PSM) analyses. Using 1:1 nearest-neighbor matching with a caliper of 0.1, the participants for each pattern (C1–C4) were matched to controls without the pattern based on sociodemographic (gender, age, education, marital status, residence, and income) and behavioral factors (smoking, alcohol, and physical activity). Propensity score distributions ranged from 0.11–0.57 (C1) and 0.02–0.28 (C2/C3) to 0.02–0.33 (C4). The results from matched cohorts reinforced our primary findings: the average treatment effect on the treated (ATT) was highest for C4 (ATT = 0.329, 95% CI: 0.287–0.373), followed by C2/C3 (ATT = 0.161, 95% CI: 0.110–0.212) and C1 (ATT = 0.099, 95% CI: 0.078–0.121). Post-matching logistic regression yielded odds ratios closely aligned with the fully adjusted model (Model 3)—C1: OR = 1.55 (1.41–1.71) vs. 1.65 (1.52–1.80); C2: OR = 1.97 (1.59–2.44) vs. 2.24 (2.05–2.45); C3: OR = 1.97 (1.59–2.44) vs. 2.19 (1.87–2.56); C4: OR = 3.93 (3.21–4.82) vs. 4.56 (3.90–5.34)—demonstrating consistent directionality, significance, and effect magnitude despite sample size reduction after matching.

To evaluate potential bias from self-reported disease misclassification, we conducted sensitivity analyses informed by CHARLS biomarker validation studies. Based on established false-negative and false-positive rates from prior CHARLS waves, we systematically reclassified participants by converting randomly selected subsets of self-reported non-cases to cases [14]. Latent class analysis on this adjusted dataset yielded nearly identical results to our primary analysis: the 5-class solution remained optimal with minimal fit statistic deviations (AIC: 176,630.5, BIC: 177,244, entropy: 0.737). Crucially, the clinical interpretability of all patterns—particularly the high-risk cardio-metabolic class (C4)—remained unchanged. This consistency suggests that under reporting of asymptomatic conditions, while non-negligible, does not materially alter the identified multimorbidity patterns or their depression associations.

To address potential biases from missing data, we implemented multiple imputations using chained equations (MICE) with 20 imputations, including all demographic (gender and age), socioeconomic (education, marital status, residence, and annual household income), behavioral (smoking status, alcohol consumption, and physical activity), and disease variables in the imputation model. Complete latent class analysis (LCA), logistic regression, and XGBoost analyses were conducted on each imputed dataset, with the results pooled via Rubin’s rules. A sensitivity analysis was used to compare these outcomes with the original complete-case analysis (excluding missing values). Despite using different missing data strategies, the LCA method retained five comorbidity patterns, which is consistent with prior findings. The logistic regression on imputed data showed significant associations for all non-reference patterns (C1, OR = 1.46, 95% CI: 1.35–1.58; C2, OR = 2.34, 95% CI: 2.15–2.56, C3, OR = 2.16, 95% CI: 1.77–2.64; C4, OR = 3.64, 95% CI: 3.14–4.22), mirrored by XGBoost models. These results aligned with the complete-case analyses, confirming robust associations between multimorbidity and depression across both methods.

## 4. Discussion

In this study, the complex associations between multimorbidity patterns and depression were systematically revealed, along with their predictive mechanisms. Through latent class analysis (LCA), four distinct multimorbidity patterns (Class 1–Class 4) were identified, all of which demonstrated significant independent associations with depression risk (OR values ranging from 1.65 to 4.56). Among these, Class 4, dominated by cardiovascular–digestive comorbidities (OR = 4.56), exhibited the strongest independent effect, suggesting that higher pathological complexity corresponds to a more pronounced increase in depression risk. Further analysis using the XGBoost model and SHAP values revealed that all four multimorbidity patterns positively predicted depression, though their contributions varied: Class 4 demonstrated the strongest direct effect on depression, while Class 2 showed the most prominent overall contribution within the model, with its broad and significant influence indicating potential multifactorial pathophysiological or psychosocial mechanisms linking this multimorbidity pattern to depression. In contrast, the predictive effects of Class 1 and Class 3 were relatively weaker, with Class 3 displaying the lowest overall contribution among the four patterns. Notably, sociodemographic variables—including income level, educational level, gender, and rural/urban residential region—emerged as core predictors in the model and exhibited significant synergistic effects with multimorbidity patterns. These findings suggested that the impact of multimorbidity on depression may be embedded within the interplay of individual socioeconomic characteristics and health environments. By integrating traditional statistical methods with the machine learning model, this study not only validated the hypothesis of multimorbidity as an independent risk factor for depression but also highlighted predictive efficacy differences across refined multimorbidity classifications. These results provide empirical evidence for identifying high-risk populations and developing stratified intervention strategies.

The findings of this study align with prior evidence demonstrating a dose–response relationship between the complexity of chronic disease multimorbidity and depression risk [15]. For instance, cross-sectional analyses consistently indicate a higher prevalence of depression among individuals with three or more chronic conditions compared to those with a single disease, while longitudinal studies confirm bidirectional associations between depression and conditions such as cardiovascular and digestive diseases [16,17,18,19,20]. However, existing research predominantly quantifies multimorbidity through simple disease counts or pairwise combinations, neglecting pathophysiological clustering characteristics. Notably, limited studies have investigated the associations between multimorbidity patterns and depression, despite their critical implications for early clinical intervention. Such research could enable clinicians to implement targeted preventive measures to mitigate depression onset or progression, while also facilitating the elucidation of underlying mechanisms driving disease development.

Through latent class analysis (LCA), four distinct multimorbidity patterns were identified, with the cardiovascular-dominant Class 4 emerging as a strong independent risk factor for depression (OR = 4.56). This finding aligns with prior studies confirming robust associations between cardiovascular diseases and depression [21,22]. The heightened depression risk observed in the cardiovascular–digestive multimorbidity cluster (Class 4) may stem from synergistic interactions across inflammatory, autonomic nervous, and metabolic pathways shared by these two systems [22,23,24,25]. Furthermore, patients in this class face compounded burdens, including polypharmacy, frequent healthcare utilization, and substantial treatment costs. These multifaceted stressors exacerbate psychological distress, predisposing individuals to anxiety and depression [26].

Both regression analysis and XGBoost modeling demonstrated that Class 3 (respiratory-dominant cluster) exhibited relatively weaker association with depression and limited predictive utility for depressive outcomes. These findings align with a previous study investigating the correlation between bronchial asthma and psychological disorders, which reported no significant association between pulmonary function indices and depressive symptoms [27]. This phenomenon may be attributed to the relatively well-elucidated pathological mechanisms of respiratory diseases, which reduce psychological distress associated with etiological ambiguity and foster stronger patient confidence in disease management. Moreover, the prevalence of the respiratory comorbidity group was the lowest among the four multimorbidity groups, and its smaller sample size is considered to be one of the reasons leading to its lower predictive efficacy [28].

Notably, the machine learning framework uncovered novel mechanistic insights obscured by traditional regression models [29]. While logistic regression identified the cardiovascular–digestive multimorbidity pattern (Class 4) as having the strongest independent association with depression, XGBoost-SHAP analysis revealed that the digestive–joint pattern (Class 2) exhibited the highest overall predictive contribution to depression risk. This discrepancy may stem from two key factors. First, logistic regression quantifies independent effects under linear assumptions, while XGBoost captures nonlinear interactions. In particular, the significant synergistic effect between Class 2’s pattern and female gender (interaction gain = 0.115) may enhance the overall predictive efficacy of Class 2 for depression [30,31]. Second, the prevalence-mediated predictive modulation likely plays a role: the digestive–joint pattern demonstrates higher population prevalence and broader societal applications compared to the cardio-digestive pattern, amplifying its population-level impact. In contrast, Class 4 represents a specialized high-risk subgroup with narrower sociodemographic influence. The interaction between the digestive–joint multimorbidity pattern and female gender may be attributed to the fact that articular disorders are often accompanied by chronic pain symptoms [32]. Studies have shown that women with chronic pain conditions are at a significantly higher risk of depression than men [33]. This phenomenon may be attributed to the fact that estrogen in women can enhance central nervous system sensitivity to pain, thereby inducing depression, and women’s tendency to repeatedly dwell on the negative impacts of pain closely overlaps with core depressive symptoms such as feelings of hopelessness and difficulty concentrating [34,35,36,37].

This study further identified significant synergistic effects between various sociodemographic factors and depression risk across distinct multimorbidity groups. Specifically, lowest income and rural residency demonstrated strong interactions with most multimorbidity clusters, indicating that they are high-risk factors for most of the multimorbid population. This phenomenon may be linked to systemic deficiencies in chronic disease management resources in rural areas, and suggests that living standards and the environment are closely associated with people’s depressive emotions [38,39]. Furthermore, the Class 3 multimorbidity pattern exhibited elevated depression risk among individuals with lower educational attainment, suggesting that limited cognitive resources may exacerbate its pathophysiological mechanisms. Future research should investigate the potential mediating role of health literacy in this association [40,41]. Notably, the most pronounced interaction emerged between female sex and the Class 2 multimorbidity pattern (Gain = 0.115), highlighting the urgent need for gender-tailored interventions targeting chronic pain management and caregiving burden mitigation in this population.

The findings of this study can inform depression screening and early intervention for patients with chronic diseases. We recommend establishing a stratified intervention framework for depression, integrating the PHQ-9 depression screening into rural primary care chronic disease management programs, with prioritization for individuals with over 2 comorbidities. Low-income individuals with chronic diseases should also be prioritized as a key target population for depression screening. Routine depression protocols are advised for all patients with cardiovascular–digestive diseases, while female patients with digestive–joint diseases are suggested to be prioritized for optimized depression screening. This stratified management system allows for the targeted allocation of limited healthcare resources to intervention points with the strongest predictive efficacy.

To our knowledge, this study represents a pioneering exploration of the associations between distinct multimorbidity patterns and depression, which has a certain clinical translation value and implications for optimizing chronic disease management. From a methodological perspective, a multi-method framework integrating latent class analysis (LCA), logistic regression, and interpretable machine learning effectively addresses two major limitations of current comorbidity research: whether multimorbidity patterns are associated with depression and the strength of their predictive effects. Although multiple previous studies have applied various clustering analysis methods to identify comorbidity patterns, their focus has mostly been limited to the associations with physical functions (such as disability rates and hospitalization risks), and the predictive role of these patterns in mental health has often been overlooked. This study elucidates the underlying mechanisms through which distinct multimorbidity patterns influence depression.

However, some limitations need to be acknowledged. First, the interpretive complexity arising from divergent findings between associative and predictive metrics highlights a fundamental challenge in translating multimorbidity research into practice. While this reflects legitimate methodological distinctions (biological risk intensity vs. population-level predictive utility), it necessitates dual reporting of both metrics to guide clinical actions: ORs for targeting high-risk subgroups, and SHAP for optimizing population screening efficiency. Second, it is not possible to establish causal relationships between multimorbidity patterns and depression using cross-sectional data. Third, primary reliance on CHARLS—a China-centric cohort—and predominant citation of local studies may constrain direct generalizability to global populations where healthcare systems, comorbidity distributions, and social determinants of depression exhibit certain differences. Fourth, the cross-sectional design precludes the assessment of competing mortality risks, and incomplete longitudinal data on chronic diseases limit dynamic comorbidity analyses. Future investigations are suggested to consider implementing multi-wave dynamic tracking designs to validate causal pathways.

## 5. Conclusions

Overall, this study revealed significant independent associations between all four multimorbidity patterns and depression risk, among which include cardio-metabolic patterns, digestive–joint patterns, respiratory patterns, and cardiovascular–digestive patterns. The cardiovascular–digestive cluster demonstrated the strongest association with depression risk. However, the digestive–joint pattern exhibited the strongest predictive utility for depression among all multimorbidity configurations, attributable to its complex nonlinear interactions and higher population prevalence. The cardio-metabolic and cardiovascular–digestive clusters demonstrated lower predictive performance, while the respiratory cluster showed comparatively limited predictive capacity. Furthermore, socioeconomic determinants including income level, educational level, gender, and rural/urban residence emerged as key predictors of depression, and demonstrated significant interaction effects with multimorbidity patterns.

This innovative framework not only provides a scientific basis for identifying elderly patients with multimorbidity at higher risk of depression in clinical practice, but also offers an evidence-based roadmap for public health policymakers to design stepped intervention strategies. It is recommended that research efforts be directed towards exploring precision psychological interventions customized to comorbidity profiles and promoting the transformation of chronic disease management towards an integrated care paradigm that comprehensively addresses both physical and mental health.

## Figures and Tables

**Table 1:** Baseline characteristics of participants by depression status (N = 17,424).

Variables	Total (*n* = 17,424)	Depression (6543)	Non-Depression (10,881)	$\chi^{2}$ Value	*p* Value	Q Value
Age (years)				89.78	<0.001	<0.05
45–59	7207 (41.36)	2428 (37.11)	4779 (43.92)			
60–69	5836 (33.50)	2268 (34.66)	3568 (32.79)			
70 and above	4381 (25.14)	1847 (28.23)	2534 (23.29)			
Gender				457.407	<0.001	<0.05
Male	8141 (46.72)	2375 (36.30)	5766 (53.00)			
Female	9283 (53.28)	4168 (63.70)	5115 (47.00)			
Marital status				140.037	<0.001	<0.05
Married	14,881 (85.41)	5321 (81.32)	9560 (87.86)			
Other	2543 (14.59)	1222 (18.68)	1321 (12.14)			
Educational level				602.927	<0.001	<0.05
Incomplete primary education	7380 (42.36)	3443 (52.62)	3937 (36.18)			
Primary education	3879 (22.26)	1452 (22.19)	2427 (22.30)			
Secondary education	3923 (22.51)	1156 (17.67)	2767 (25.43)			
Higher education	2242 (12.87)	492 (7.52)	1750 (16.09)			
Place of residence				291.789	<0.001	<0.05
Non-rural	6875 (39.46)	2048 (31.30)	4827 (44.36)			
Rural	10,549 (60.54)	4495 (68.70)	6054 (55.64)			
Physical activity				10.45	0.001	<0.05
No	1711 (9.82)	704 (10.76)	1007 (9.25)			
Yes	15,713 (90.18)	5839 (89.24)	9874 (90.75)			
Drinking				258.783	<0.001	<0.05
No	11,073 (63.55)	4653 (71.11)	6420 (59.00)			
Yes	6351 (36.45)	1890 (28.89)	4461 (41.00)			
Smoking				106.555	<0.001	<0.05
No	12,941 (74.27)	5148 (78.68)	7793 (71.62)			
Yes	4483 (25.73)	1395 (21.32)	3088 (28.38)			
Income quintiles				577.096	<0.001	<0.05
Lowest-income quintile	3486 (20.00)	1698 (25.95)	1788 (16.43)			
Lower-income quintile	3455 (19.83)	1547 (23.64)	1908 (17.54)			
Middle-income quintile	2834 (16.26)	1027 (15.70)	1807 (16.61)			
Upper-income quintile	4164 (23.90)	1459 (22.30)	2705 (24.86)			
Highest-income quintile	3485 (20.01)	812 (12.41)	2673 (24.56)			
Multimorbidity				595.418	<0.001	<0.05
No	7323 (42.03)	1980 (30.26)	5343 (49.10)			
Yes	10,101 (57.97)	4563 (69.74)	5538 (50.90)			
Multimorbidity patterns				799.085	<0.001	<0.05
Pattern1	3779 (21.69)	1511 (23.09)	2268 (20.84)			
Pattern2	2835 (16.27)	1420 (21.70)	1415 (13.00)			
Pattern3	738 (4.24)	355 (5.43)	383 (3.52)			
Pattern4	838 (4.81)	555 (8.48)	283 (2.60)			
Pattern5	9234 (52.99)	2702 (41.30)	6532 (60.04)			

Note: *p*-value less than 0.05 was defined as significant, and FDR-adjusted Q value less than 0.05 was defined as significant.

**Table 2:** The associations between multimorbidity patterns and depression.

Variable	Model 1				Model 2				Model 3		
	OR (95% CI)	*p*-Value	Q Value		OR (95% CI)	*p*-Value	Q Value		OR (95% CI)	*p*-Value	Q Value
Cardio-Metabolic	1.61	<0.001	<0.05		1.65	<0.001	<0.05		1.65	<0.001	<0.05
	(1.49–1.74)			(1.52–1.80)			(1.52–1.80)				
digestive–joint	2.43	<0.001	<0.05		2.25	<0.001	<0.05		2.24	<0.001	<0.05
	(2.23–2.64)			(2.06–2.46)			(2.05–2.45)				
Respiratory	2.24	<0.001	<0.05		2.2	<0.001	<0.05		2.19	<0.001	<0.05
	(1.93–2.61)			(1.88–2.57)			(1.87–2.56)				
Cardio-Digestive	4.74	<0.001	<0.05		4.58	<0.001	<0.05		4.56	<0.001	<0.05
	(4.08–5.51)			(3.92–5.36)			(3.90–5.34)				
Relatively Healthy	1.00 (ref)	—		1.00 (ref)	—		1.00 (ref)	—			

Model 1: non-adjusted (univariate analysis). Model 2: demographic and socioeconomic variables (gender, age, education level, marital status, residence, and annual household income) were adjusted. Model 3: Model 2 plus adjusted behavior factors (smoking status, alcohol consumption, and physical activity). OR values were referenced to Class 5. *p*-value less than 0.05 was defined as significant and FDR-adjusted Q value less than 0.05 was defined as significant. OR=exp(β), where β represents regression coefficient. 95%CIforOR=exp(β±1.96×SE(β)), where SE(β) represents the standard error of β.

## Data Availability

The data utilized in this analysis are publicly accessible through the China Health and Retirement Longitudinal Study (CHARLS), hosted by Peking University (http://charls.pku.eud.cn/en).

## Appendix A

**Table A1:** Model fit statistics for latent class analysis models specifying one to six classes.

Model	AIC	BIC	SSA-BIC	Entropy	LMR-LRT	BLRT
C1	188,769.70	188,886.18	188,838.51	-	-	-
C2	179,474.79	179,715.52	179,617.00	0.649	<0.001	<0.001
C3	177,538.82	177,903.80	177,754.44	0.679	<0.001	<0.001
C4	176,544.54	177,033.78	176,833.57	0.732	<0.001	<0.001
C5	175,729.93	176,343.41	176,092.35	0.66	<0.001	<0.001
C6	174,949.96	175,687.69	175,385.79	0.704	<0.001	<0.001

## Appendix B

**Table A2:** The association between multimorbidity and depression.

Model	b	SE	Wald $\chi^{2}$	*p*-Value	OR (95% CI)
Model 1	0.799	0.033	584.705	<0.001	2.223 (2.084–2.372)
Model 2	0.77	0.035	490.456	<0.001	2.159 (2.017–2.311)
Model 3	0.768	0.035	486.244	<0.001	2.156 (2.014–2.309)

Model 1: non-adjusted (univariate analysis). Model 2: demographic and socioeconomic variables (gender, age, education level, marital status, residence, annual household income) were adjusted. Model 3: Model 2 as well as behavior factors (smoking status, alcohol consumption, physical activity) were adjusted.
